# Downregulation of miR-224-5p Promotes Migration and Proliferation in Human Dental Pulp Stem Cells

**DOI:** 10.1155/2019/4759060

**Published:** 2019-07-18

**Authors:** Zhihong Ke, Zailing Qiu, Tingting Xiao, Jianchai Zeng, Luning Zou, Xuemei Lin, Xuegang Hu, Shihan Lin, Hongbing Lv

**Affiliations:** ^1^Department of Endodontics and Operative Dentistry, School and Hospital of Stomatology, Fujian Medical University, Fuzhou 350000, China; ^2^Key Laboratory of Stomatology (Fujian Medical University), Fujian Province University, Fuzhou 350000, China; ^3^Fujian Provincial Engineering Research Center of Oral Biomaterial, Fuzhou 350000, China

## Abstract

**Introduction:**

Pulp regeneration, as a treatment for pulp necrosis, has significant advantages over root canal therapy for the preservation of living pulp. To date, research on pulp regeneration has mainly focused on the transplantation of pulp stem cells into the root canal, but there is still a lack of research on the migration of pulp cells into the root canal via cell homing. Stem cells from the apical tooth papilla (SCAP) are recognized as multidirectional stem cells, but these cells are difficult to obtain. MicroRNAs are small noncoding RNAs that play crucial roles in regulating normal and pathologic functions. We hypothesized that some types of microRNAs might improve the migration and proliferation function of dental pulp stem cells (DPSCs), which are easily obtained in clinical practice, and as a result, DPSCs might replace SCAP and provide valuable information for regenerative endodontics.

**Methods:**

Magnetic activated cell sorting of DPSCs and SCAP was performed. Next-generation sequencing was performed to examine DPSCs and SCAP miRNAs expression and to identify the most significant differentially expressed miRNA. CCK-8 and transwell assays were used to determine the impact of this miRNA on DPSCs proliferation and migration.

**Results:**

The most significant differentially expressed miRNA between DPSCs and SCAP was miR-224-5p. Downregulating miR-224-5p promoted DPSCs proliferation and migration; the opposite results were observed when miR-224-5p was upregulated.

**Conclusion:**

MiR-224-5p promotes proliferation and migration in DPSCs, a finding that is of great significance for further exploring the role of dental pulp stem cells in regenerative endodontics.

## 1. Introduction

Human dental pulp stem cells (DPSCs) and stem cells from the apical papilla (SCAP) have been regarded as a very valuable resource for dental tissue regeneration [1]. These two types of cells originate from different tissues: DPSCs from dental pulp and SCAP from the apical papilla. Both types of cells are odontogenic stem cells. As with other mesenchymal stem cells, DPSCs and SCAP have the potential for high proliferation, self-renewal, and multidirectional differentiation. After induction, DPSCs and SCAP can differentiate into nervus, lipids, and dentin. Although studies have reported that SCAP have a significantly higher proliferation rate and mineralization potential than DPSCs [2, 3], apical papilla tissues are very difficult to collect compared to dental pulp. In other words, DPSCs are more convenient to obtain than are SCAP. MicroRNAs (miRNAs) are a type of noncoding RNA molecule approximately 19-22 nucleotides in length with important functions in animals through the targeting of mRNAs, leading to cleavage or translational repression [4–7]. Lentivirus-mediated RNAi has been widely used to alter gene expression in mammalian teeth [8]. Previous approaches have focused on testing the osteogenic/dentinogenic function of DPSCs without considering the cell migration that would need to occur to reach the site where the cells are needed [9, 10]. From a clinical perspective, cell migration to the dentin wall is as important as the potential of the stem cells. If the cells cannot reach the dentin wall, the formation of a healthy pulp-dentin complex is not possible. To date, there is a lack of evidence demonstrating which miRNA can significantly enhance the migration ability of dental pulp stem cells. Therefore, we tested differential miRNAs expression between DPSCs and SCAP and predicted the function of a selected miRNA. We attempted to identify the key miRNA that drives SCAP stemness to enhance stemness functions in DPSCs.

## 2. Materials and Methods

### 2.1. Cell Culture

Dental pulp and apical tooth papilla tissue were extracted under sterile conditions from healthy third molars of 17- to 20-year-old adults. Tissues were washed twice with sterile phosphate-buffered saline (PBS; 0.01 M, pH = 7.4) supplemented with antibiotics (100 U/ml penicillin and 100 U/ml streptomycin). The tissues were cut into pieces and digested with CD enzyme (type I collagenase at 3 mg/ml and dispase II enzyme at 4 mg/ml at 37°C for 30 min). After the digestion was terminated, the cells were centrifuged at 1,000 r/min for 5 min (high-speed bench centrifuge, Eppendorf, Germany). A single-cell suspension was obtained by filtering the cells. The cells were transferred to 100-mm Petri dishes (Corning, USA) in *α*-MEM (HyClone, USA) supplemented with 10% fetal bovine serum (HyClone, USA), 100 U/ml penicillin, and 100 U/ml streptomycin. All cultures were incubated at 37°C with 5% CO_2_ for 7 to 10 days. When the cell confluence reached 70%~80%, trypsin (Gibco, USA) was used to digest the cells for passaging; these cells were considered the first generation, and the second generation of cells was used for ensuing experiments.

### 2.2. Magnetic Activated Cell Sorting and Identification

For cell-sorting experiments, 1×10^7^ dental pulp cells and apical papilla cells at the third passage were resuspended in human STRO-1 monoclonal antibody (R&D Systems, USA) with 60 *μ*l running buffer (R&D Systems, USA), 20 *μ*l FcR blocking (R&D Systems, USA), and 20 *μ*l magnetic activated cell sorting (Miltenyi Biotec, Germany) solutions and preincubated for 15 min at 4°C. The cell suspension was applied to the column, and the flow-through containing unlabeled cells was collected. STRO-1(+) cells were expanded.

### 2.3. Osteogenic Differentiation

The STRO-1(+) cells, including DPSCs and SCAP, were digested with trypsin and planted at 5×10^4^ cells/well in 6-well plates. When the two types of cells confluence reached 70%~80%, the standard medium was replaced with Mesenchymal Stem Cell Osteogenic Differentiation Medium (HUXMA-90021, Cyagen, USA) for 21 days. After differentiation, the cells were stained according to the instructions of an ALP detection kit (Nanjing Jiancheng Biotechnology Institute, China) and Alizarin red (Nanjing Senbeja Biological Technology Co., Ltd., China).

### 2.4. Next-Generation Sequencing for miRNA Expression Profiles

DPSCs and SCAP were mixed with Trizol for extraction of total RNA. The purified RNA was analyzed using a Bioanalyzer 2100. Libraries were constructed using a TruSeq Small RNA Sample Prep Kit (Illumina, USA) according to the manufacturer's instructions, which involved PCR amplification after the ligation of 3' and 5' adapters. Enriched regions were analyzed, and the types of small RNAs were classified. Gene Ontology and miRTarBase were used to predict target genes and related functions.

### 2.5. Reverse Transcription and Quantitative Real-Time PCR

Quantitative real-time PCR was carried out with SYBR Premix Ex Taq II (Tli RNaseH Plus, Takara, Japan). cDNA was synthesized using a Mir-X miRNA First-Strand Synthesis Kit (Takara, Japan). The fold change of miR-224-5p expression in DPSCs versus SCAP was calculated with the 2^−ΔΔCt^ method. The experiment was repeated 3 times.

### 2.6. Dental Pulp Stem Cells Transfection

A miR-224 mimic (product ID, 19417-1), miR-224 mimic negative control (NC) (product ID, LVCON238), miR-224 inhibitor (product ID, 24681-1), and miR-224 inhibitor negative control (product ID, LVCON137) were synthesized by Genechem (Shanghai Genechem, China).

Dental pulp stem cells were digested with trypsin and planted at 5×10^4^ cells/well in 6-well plates. Transduction of the DPSCs was performed by exposing them to dilutions of the viral supernatant in the presence of HitransG P (Shanghai Genechem, China) for 12 h and then changing the dilutions to standard medium for 48 h. Next, 2 *μ*g/ml puromycin (Shanghai Genechem, China) was added to kill untransfected cells that were not fluorescently labeled. Finally, cells were collected for experiments.

### 2.7. Proliferation and Migration

Cell proliferation was measured for 6 days using a Cell Counting Kit-8 (Dojindo, Japan), and the samples were measured at 450 nm. The experiment was repeated 3 times.

Cells were serum starved for 12-24 h, digested, and centrifuged at 1,000 r/min for 5 min. A 1×10^5^ cell/ml cell suspension was prepared. A total of 200 *μ*l of cell suspension was added to the upper chamber of a transwell chamber (Transwell BD Matrigel, 3422, Costar, USA), and 500 *μ*l of culture medium containing FBS was added to the lower chamber. After culture for 24 h, crystal violet was added for staining, and the cells were counted and photographed under as inverted microscope. The experiment was repeated 3 times.

### 2.8. Statistical Analysis

Statistical significance between groups was determined by a two-tailed Student's* t*-test. The association between miRNA expression and proliferation characteristics was analyzed using repeated ANOVA. A* P* value of <0.05 was considered significant. All statistical analyses were performed with SPSS Statistics version 23.0 (IBM, Armonk, USA).

## 3. Results

### 3.1. DPSCs and SCAP Isolation and Identification

**Figure 1:**
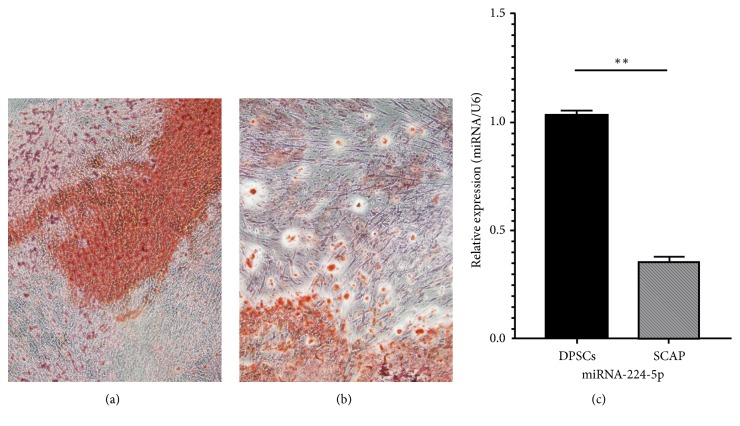
Osteogenic differentiation for 21 days. Red areas are calcified nodules. (a) Stem cells from the apical papilla (SCAP). (b) Human dental pulp stem cells (DPSCs). There are more calcified nodules in picture (a) than in picture (b). Original magnification: 40×. (c) Quantitative RT-PCR showing the levels of miR-224-5p expression in DPSCs and SCAP. Values are expressed as relative expression with respect to the endogenous control gene U6 (2^−ΔΔCt^). Data represent the mean±SEM of 3 independent experiments conducted in duplicate, *∗∗*P<0.01.

We performed a cell-sorting experiment using STRO-1 to purify DPSCs and SCAP. The cells underwent osteogenic differentiation for 21 days, and the differentiation ability of the DPSCs and SCAP was evaluated. The cells were identified with an ALP detection kit and Alizarin red staining. SCAP showed more osteogenic potential and higher proliferation than did DPSCs (Figure 1).

### 3.2. Next-Generation Sequencing Results and Functional Prediction

To explore differential expression of miRNAs between DPSCs and SCAP, we used the R package DESeq2 (version 1.6.1) and selected miRNAs with expression fold-differences >1.5 (P<0.05) from each group of data. The results revealed 7 significant differentially expressed miRNAs, including miR-224-5-p, miR-1247-5p, miR-3065-3p, miR-452-3p, miR-767-5p, miR-4284, and miR-146a-5p, among which expression of miR-224-5p was the most significantly different between the groups.

**Figure 2:**
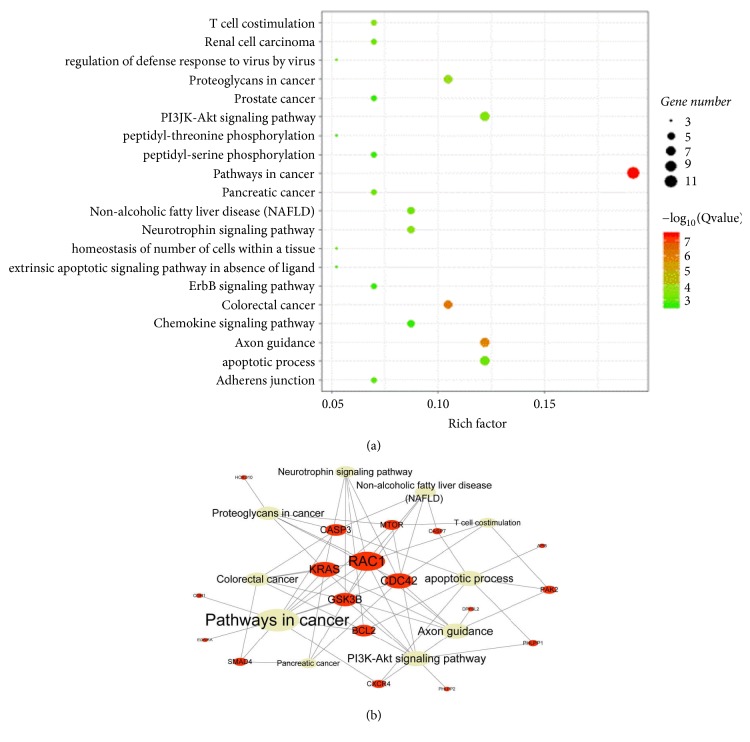
(a) The size of the bubbles represents the number of target genes, and the color of bubbles indicates the degree of correlation. When the color is closer to red, the correlation is higher. (b) The red node represents the target genes of miR-224-5p, the gray node represents the pathway of those target genes, and the node size represents connectivity. The larger the target gene node is, the more functions it covers. The larger the pathway node is, the more target genes it covers.

Based on miRTarBase, highly reliable miR-224-5p-related target genes were identified. Gene function and Gene Ontology, i.e., biological process, cellular component, and molecular function, analyses were performed. The results showed enrichment of the top 20 pathways mainly in the cancer pathway, PI3K-AKT signaling pathway, and apoptotic process. These pathways are related to the regulation of cell proliferation, apoptosis, and pluripotency (Figure 2).

### 3.3. RNA Isolation and Quantitative Real-Time PCR

We carried out qRT-PCR to examine miRNA expression in DPSCs and SCAP. miR-224-5p was downregulated in SCAP compared to DPSCs (Figure 1(c)).

### 3.4. Transfection Efficiency of Lentivirus Expressing miR-224-5p

**Figure 3:**
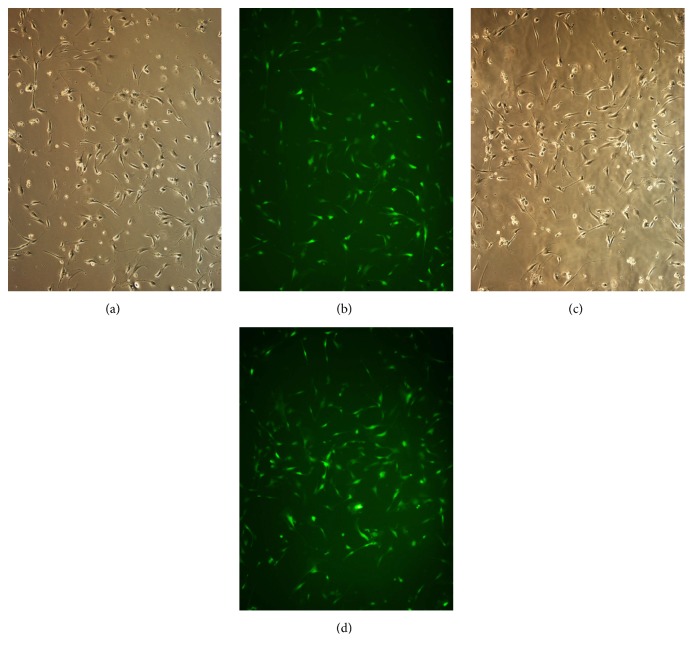
Transfection efficiency of the miR-224-5p mimic (a, b) and inhibitor (c, d) in human dental pulp stem cells. (a, c) Dental pulp stem cells under bright-field microscopy. (b, d) Dental pulp stem cells under fluorescence microscopy. The transfection efficiency was 80-90%. Original magnification: 100×.

The lentivirus transfection efficiency was evaluated based on the fluorescence of transfected cells. When the cells were successfully infected, they could be observed by fluorescence microscopy. We grouped the experimental conditions to increase the transfection efficiency (Figure 3).

### 3.5. Effects of miR-224-5p Up-/Downregulation on the Proliferation and Migration of DPSCs

**Figure 4:**
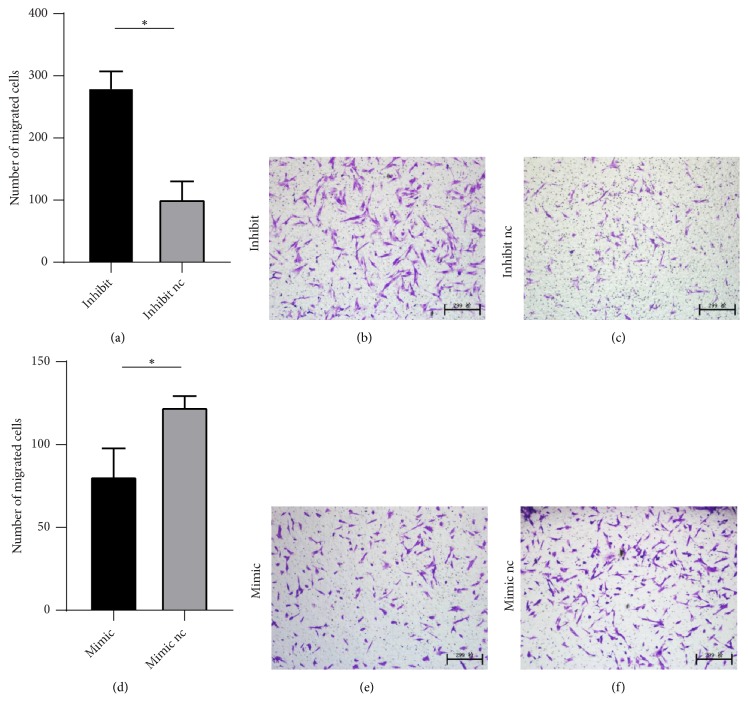
Effects of miR-224-5p expression on dental pulp stem cell migration. (a–c) Downregulation of miR-224-5p significantly increased DPSCs migration. *∗*P<0.05, compared with inhibitor nc cells. (d–f) Overexpression of miR-224-5p significantly decreased DPSCs migration; *∗*P<0.05, compared with mimic nc. Original magnification: 100×.

**Figure 5:**
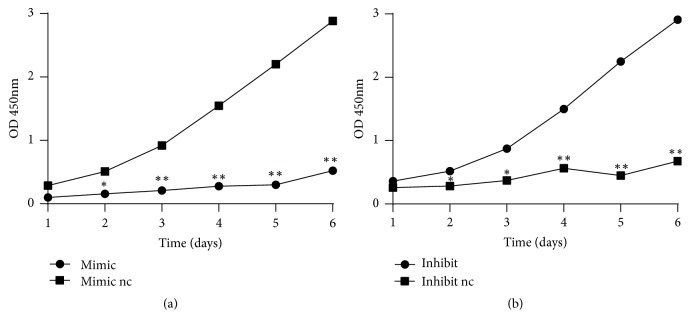
Effects of miR-224-5p expression on dental pulp stem cell proliferation. (a) Overexpression of miR-224-5p significantly decreased DPSCs proliferation; *∗*P<0.05, *∗∗*P<0.01, compared with mimic nc. (b) miR-224-5p downregulation significantly increased DPSCs migration. *∗*P<0.05, *∗∗*P<0.01 compared with inhibitor nc.

We observed DPSCs in upregulated (mimics) and control groups (mimics nc) and in downregulated (inhibitor) and control groups (inhibitor nc). We found that, compared to the controls, downregulation of miR-224-5p enhanced DPSCs migration (Figure 4) and proliferation (Figure 5); in contrast, miR-224-5p upregulation decreased DPSCs stemness.

## 4. Discussion

We obtained DPSCs and SCAP from dental pulp tissues and dental apical tissues, respectively. Previous studies have demonstrated that these stem cells are STRO-1 positive [11, 12]. Thus, STRO-1 has been regarded as one of the markers of these stem cells. In this study, we used STRO-1 to separate DPSCs and SCAP from dental pulp cells and apical papilla cells. Through osteogenic differentiation, SCAP produced more calcified nodules compared with DPSCs. These results were in line with the research of Bakopoulou et al., in which SCAP showed a significantly higher mineralization potential than DPSCs [2]. The results of next-generation sequencing showed that miR-224-5p was the most significantly differentially expressed of seven miRNAs. The target genes of this miRNA suggest that miR-224-5p may be closely related to cell proliferation, migration, and angiogenesis. Quantitative RT-PCR validated the results of sequencing. Furthermore, overexpression of miR-224-5p in DPSCs decreased migration and proliferation compared to those in the control. When miR-224-5p was downregulated, DPSCs migration and proliferation were enhanced compared to those in the control.

The traditional management of pulpitis is root canal treatment (RCT), which involves cleaning, shaping, and filling the root canal to control inflammation. The success rate of RCT is 68%-85% [13], and the rate is increasing with the development of the NiTi instrument. However, due to a lack of vital pulp, tooth tissue is lost as a result of nutrition and reparative dentin. Thus, some complications, such as postoperative fractures and reinfections, occur frequently. Overall, this technique is not ideal for preserving teeth. Iwaya SI [14] first proposed the concept of pulp revascularization in 2001, which is now referred to as pulp regeneration therapy.

As an alternative method, regenerative endodontics replaces necrotic pulp tissue with regenerated pulp-like tissue, with replanted dental pulp cells [15, 16] or cell homing [17–19]. Many studies have been carried out to determine the mechanisms and methods for transplanting dental pulp stem cells to the root canal, including studies at the cellular level and animal experiments [11, 20]. Compared to the transplantation of stem cells, promoting cell homing appears to be a more economic and convenient way to achieve the desired effects. Research has shown that transplantation requires the isolation and manipulation of stem cells in vitro. However, it is possible to contaminate the cells, leading to the failure of regeneration of the dental pulp [12]. We designed this study to determine which miRNA can significantly enhance the migration and proliferation of DPSCs. Furthermore, we sought to determine those miRNAs that might promote dental stem cell homing.

Due to the high multidirectional differentiation potential of stem cells from the apical tooth papilla, current research has mainly focused on inducing SCAP differentiation [1, 21–23]. However, these cells are more difficult to isolate than DPSCs. If we can enhance the differentiation potential of DPSCs, they might be considered as possible alternatives for regenerative medicine. Thus, the first problem that needs to be solved is how to improve the differentiation function of DPSCs.

To determine the reasons for the differences between these cell types, we performed sequencing and constructed miRNA differential expression profiles. Compared with other traditional sequencing methods, the advantage of next-generation sequencing is the ability to sequence genomes on a much larger scale [24]. However, the associated error rates (~0.1–15%) are also higher than are those of traditional methods [25]. Based on next-generation sequencing, we found 7 genes that were significantly (more than 1.5-fold) differentially expressed in SCAP compared with DPSCs, among which miR-224-5p was the most significantly different. To further verify the sequencing results, we conducted quantitative RT-PCR, and the results were consistent with those of sequencing.

MicroRNAs are small noncoding RNAs that play crucial roles in regulating normal and pathologic functions. It is now clear that miRNAs play important roles in directing stem cell fate by fine-tuning the levels of various factors [26].

Expression of miR-224 can activate signaling pathways and lead to tumor cell proliferation, metastasis, and occurrence, such as in prostate cancer [27] and pancreatic cancer [28]. Additionally, miR-224 can affect normal cell migration, such as SW80 cells [29] and ameloblasts [30].

According to the results of GO analysis, KEGG pathway analysis, and target gene prediction, we preliminarily determined the top 20 pathways to be mainly enriched in the cancer pathway, PI3K-AKT signaling pathway, and apoptotic process. Some researchers have studied the PI3K-AKT signaling pathway, which affects cell proliferation and migration in cancers and is activated by CEMIP in promoting ovarian cancer tumorigenesis and progression [31]. Indeed, by targeting the PI3K-AKT signaling pathway, AAP-H showed a good antiproliferative effect on DU-145 prostate cancer cells [32]. The FN1 gene acts as a regulator of glioma cell activity, and its suppression inhibits the cell proliferation and reduces migration and invasion that occurs through activation of the PI3K/AKT signaling pathway [33]. Meanwhile, CDC42 is the most notable among target genes. CDC42 is related to many functions, and it is reported to be associated with the invasion, migration, and proliferation of gastric cancer cells [34] and cervical tumors [35].

However, there is no research focused on the effect of miR-224-5p in DPSCs. We showed that downregulating miR-224-5p expression promoted DPSCs proliferation and migration and that upregulating miR-224-5p had the opposite results. The findings are in accordance with the miR-224-5p target gene prediction.

In our research, the functions of miR-224-5p in DPSCs were discovered and verified. Nonetheless, the mechanism remains unclear. Further research must be performed on the target genes of miR-224-5p to identify a signaling pathway to promote the progression of regenerative endodontics.

## 5. Conclusion

In conclusion, our results suggest that miR-224-5p facilitates DPSCs proliferation and migration. From a clinical perspective, miR-224-5p regulation in DPSCs can potentially promote their migration to the root canal to replace damaged and necrotic pulp with new, functional cells, leading to the success of regenerative endodontics.

## Data Availability

The prediction data used to support the findings of this study have been deposited in the Figshare repository (DOI:10.6084/m9.figshare.8031689). The qRT-PCR data used to support the findings of this study have been deposited in the Figshare repository (DOI:10.6084/m9.figshare.8031686). The CCK8 data used to support the findings of this study have been deposited in the Figshare repository (DOI:10.6084/m9.figshare.8031707). The transwell data used to support the findings of this study have been deposited in the Figshare repository (DOI 10.6084/m9.figshare.8031803).

## References

[1] Nada O. A., El Backly R. M. (2018). Stem cells from the apical papilla (SCAP) as a tool for endogenous tissue regeneration. *Frontiers in Bioengineering and Biotechnology*.

[2] Bakopoulou A., Leyhausen G., Volk J. (2011). Comparative analysis of in vitro osteo/odontogenic differentiation potential of human dental pulp stem cells (DPSCs) and stem cells from the apical papilla (SCAP). *Archives of Oral Biolog*.

[3] Bakopoulou A., Leyhausen G., Volk J., Koidis P., Geurtsen W. (2013). Comparative characterization of STRO-1(neg)/CD146(pos) and STRO-1(pos)/CD146(pos) apical papilla stem cells enriched with flow cytometry. *Archives of Oral Biolog*.

[4] Bartel D. P. (2004). MicroRNAs: genomics, biogenesis, mechanism, and function. *Cell*.

[5] Chen C., Li L., Lodish H. F., Bartel D. P. (2004). MicroRNAs modulate hematopoietic lineage differentiation. *Science*.

[6] Lan H., Lu H., Wang X., Jin H. (2015). MicroRNAs as potential biomarkers in cancer: opportunities and challenges. *BioMed Research International*.

[7] Schmalz G., Li S., Burkhardt R. (2016). MicroRNAs as salivary markers for periodontal diseases: a new diagnostic approach?. *BioMed Research International*.

[8] Song Y., Zhang Z., Yu X., Yan M. (2006). Application of lentivirus-mediated RNAi in studying gene function in mammalian tooth development. *Developmental Dynamics*.

[9] Zhang P., Yang W., Wang G., Li Y. (2018). miR-143 suppresses the osteogenic differentiation of dental pulp stem cells by inactivation of NF-*κ*B signaling pathway via targeting TNF-*α*. *Archives of Oral Biolog*.

[10] Yang X., van den Dolder J., Walboomers X. F. (2007). The odontogenic potential of STRO-1 sorted rat dental pulp stem cells in vitro. *Journal of Tissue Engineering and Regenerative Medicine*.

[11] Iohara K., Imabayashi K., Ishizaka R. (2011). Complete pulp regeneration after pulpectomy by transplantation of CD105+ stem cells with stromal cell-derived factor-1. *Tissue Engineering Part A*.

[12] Ullah I., Park J., Kang Y. (2017). Transplantation of human dental pulp-derived stem cells or differentiated neuronal cells from human dental pulp-derived stem cells identically enhances regeneration of the injured peripheral nerve. *Stem Cells and Development*.

[13] Ng Y., Mann V., Rahbaran S., Lewsey J., Gulabivala K. (2007). Outcome of primary root canal treatment: systematic review of the literature – Part 1. Effects of study characteristics on probability of success. *International Endodontic Journal*.

[14] Iwaya S.-I., Ikawa M., Kubota M. (2001). Revascularization of an immature permanent tooth with apical periodontitis and sinus tract. *Dental Traumatology*.

[15] Kuo T. F., Huang A. T., Chang H. H. (2008). Regeneration of dentin-pulp complex with cementum and periodontal ligament formation using dental bud cells in gelatin-chondroitin-hyaluronan tri-copolymer scaffold in swine. *Journal of Biomedical Materials Research Part A*.

[16] Iohara K., Zheng L., Ito M. (2009). Regeneration of dental pulp after pulpotomy by transplantation of CD31^−^/CD146^−^ side population cells from a canine tooth. *Journal of Regenerative Medicine*.

[17] Albuquerque M. T. P., Valera M. C., Nakashima M., Nör J. E., Bottino M. C. (2014). Tissue-engineering-based strategies for regenerative endodontics. *Journal of Dental Research*.

[18] Laird D. J., von Andrian U. H., Wagers A. J. (2008). Stem cell trafficking in tissue development, growth, and disease. *Cell*.

[19] Chen F.-M., Wu L.-A., Zhang M., Zhang R., Sun H.-H. (2011). Homing of endogenous stem/progenitor cells for in situ tissue regeneration: promises, strategies, and translational perspectives. *Biomaterials*.

[20] Rosa V., Della Bona A., Cavalcanti B. N., Nör J. E. (2012). Tissue engineering: from research to dental clinics. *Dental Materials*.

[21] Huang G. T.-J., Sonoyama W., Liu Y., Liu H., Wang S., Shi S. (2008). The hidden treasure in apical papilla: the potential role in pulp/dentin regeneration and bioroot engineering. *Journal of Endodontics*.

[22] Wang Y., Pang X., Wu J. (2018). MicroRNA hsa-let-7b suppresses the odonto/osteogenic differentiation capacity of stem cells from apical papilla by targeting MMP1. *Journal of Cellular Biochemistry*.

[23] Koutsoumparis A., Vassili A., Bakopoulou A., Ziouta A., Tsiftsoglou A. S. (2018). Erythropoietin (rhEPOa) promotes endothelial transdifferentiation of stem cells of the apical papilla (SCAP). *Archives of Oral Biolog*.

[24] Ballester L. Y., Luthra R., Kanagal-Shamanna R., Singh R. R. (2016). Advances in clinical next-generation sequencing: target enrichment and sequencing technologies. *Expert Review of Molecular Diagnostics*.

[25] Goodwin S., McPherson J. D., McCombie W. R. (2016). Coming of age: ten years of next-generation sequencing technologies. *Nature Reviews Genetics*.

[26] Peng B., Chen Y., Leong K. W. (2015). MicroRNA delivery for regenerative medicine. *Advanced Drug Delivery Reviews*.

[27] Goto Y., Nishikawa R., Kojima S., Chiyomaru T. (2014). Tumour-suppressive microRNA-224 inhibits cancer cell migration and invasion via targeting oncogenic TPD52 in prostate cancer. *FEBS Lett*.

[28] Zhu G., Zhou L., Liu H., Shan Y., Zhang X. (2018). MicroRNA-224 promotes pancreatic cancer cell proliferation and migration by targeting the TXNIP-mediated HIF1*α* pathway. *Cellular Physiology and Biochemistry*.

[29] Liang C.-Q., Fu Y.-M., Liu Z.-Y., Xing B.-R., Jin Y., Huang J.-L. (2017). The effect of miR-224 down-regulation on SW80 cell proliferation and apoptosis and weakening of ADM drug resistance. *European Review for Medical and Pharmacological Sciences*.

[30] Fan Y., Zhou Y., Zhou X. (2015). MicroRNA 224 regulates ion transporter expression in ameloblasts to coordinate enamel mineralization. *Molecular and Cellular Biology*.

[31] Shen F., Zong Z., Liu Y., Chen S., Sheng X., Zhao Y. (2019). CEMIP promotes ovarian cancer development and progression via the PI3K/AKT signaling pathway. *Biomedicine & Pharmacotherapy*.

[32] Li X., Tang Y., Yu F. (2018). Inhibition of prostate cancer DU-145 cells proliferation by anthopleura anjunae oligopeptide (YVPGP) via PI3K/AKT/mTOR signaling pathway. *Marine Drugs*.

[33] Liao Y., Zhang Z., Zhao J., Liu J. (2018). Effects of fibronectin 1 on cell proliferation, senescence and apoptosis of human glioma cells through the PI3K/AKT signaling pathway. *Cellular Physiology and Biochemistry*.

[34] Du D. S., Yang X. Z., Wang Q., Dai W. J. (2016). Effects of CDC42 on the proliferation and invasion of gastric cancer cells. *Molecular Medicine Reports*.

[35] Ye H., Zhang Y., Geng L., Li Z. (2015). Cdc42 expression in cervical cancer and its effects on cervical tumor invasion and migration. *International Journal of Oncology*.

